# Mutations in the Two-Component GluS-GluR Regulatory System Confer Resistance to β-Lactam Antibiotics in *Burkholderia glumae*

**DOI:** 10.3389/fmicb.2021.721444

**Published:** 2021-07-26

**Authors:** Joan Marunga, Eunhye Goo, Yongsung Kang, Ingyu Hwang

**Affiliations:** ^1^Department of Agricultural Biotechnology, Seoul National University, Seoul, South Korea; ^2^Research Institute of Agriculture and Life Sciences, Seoul National University, Seoul, South Korea

**Keywords:** β-lactam resistance, β-lactamase, *Burkholderia glumae*, penicillin-binding protein, rice panicle blight, two-component systems

## Abstract

Bacteria have specific signaling systems to overcome selective pressure, such as exposure to antibiotics. The two-component system (TCS) plays an important role in the development of antibiotic resistance. Using the rice pathogen *Burkholderia glumae* BGR1 as a model organism, we showed that the GluS (BGLU_1G13350) – GluR (BGLU_1G13360) TCS, consisting of a sensor kinase and response regulator, respectively, contributes to β-lactam resistance through a distinct mechanism. Inactivation of *gluS* or *gluR* conferred resistance to β-lactam antibiotics in *B. glumae*, whereas wild-type (WT) *B. glumae* was susceptible to these antibiotics. In *gluS* and *gluR* mutants, the expression of genes encoding metallo-β-lactamases (MBLs) and penicillin-binding proteins (PBPs) was significantly higher than in the WT. GluR-His bound to the putative promoter regions of annotated genes encoding MBL (BGLU_1G21360) and PBPs (BGLU_1G13280 and BGLU_1G04560), functioning as a repressor. These results demonstrate that the potential to attain β-lactam resistance may be genetically concealed in the TCS, in contrast to the widely accepted view of the role of TCS in antibiotic resistance. Our findings provide a new perspective on antibiotic resistance mechanisms, and suggest a different therapeutic approach for successful control of bacterial pathogens.

## Introduction

Irrational use of antibiotics contributes to the emergence of antibiotic-resistant pathogens, thereby promoting disease outbreaks. Antibiotic resistance has been linked to complex bacterial systems, such as quorum sensing (Pumbwe et al., 2008; Wang et al., 2019), extra-cytoplasmic functions (ECFs), sigma factors (Yoo et al., 2016; Woods and McBride, 2017), and two-component systems (TCSs; Gooderham and Hancock, 2009; Tierney and Rather, 2019). TCSs typically comprise a sensor kinase and response regulator, and are crucial for survival and adaptation to a given environment (Hoch, 2000; Miller et al., 2004; Lingzhi et al., 2018). After perceiving an external stimulus, the sensor kinases are autophosphorylate, followed by phosphotransfer from the phosphorylated sensor kinases to the response regulators (Hoch, 2000). The phosphorylated response regulators subsequently undergo conformational modifications in order to become active, controlling the expression of target genes (Hoch, 2000). Interest in the roles of TCSs in antibiotic resistance has recently increased; TCSs are potential targets for new treatments. TCSs direct the process of antibiotic resistance through drug target modification, decreased influx, increased outflow, regulation of antibiotic-degrading enzymes, biofilm formation, and stress induction (Tierney and Rather, 2019). TCSs reported to regulate antibiotic resistance include PhoP-PhoQ of *Pseudomonas aeruginosa*, which triggers resistance to polymyxin B (McPhee et al., 2006; Gooderham and Hancock, 2009), VanS-VanR of *Enterococcus faecium* and *Streptomyces coelicolor*, which reduces affinity to vancomycin (Arthur et al., 1992; Hong et al., 2008), and CreB-CreC of *Escherichia coli* and VbrK-VbrR of *Vibrio parahaemolyticus*, which triggers β-lactam resistance through β-lactamase expression (Zamorano et al., 2014; Li et al., 2016). There have been a few reports of the resistance mechanism of *Burkholderia glumae*, a notorious pathogen that causes rice panicle blight (Kim et al., 2004; Goo et al., 2015), against antibiotics, including studies reporting the emergence of oxolinic acid-resistant strains (Hikichi et al., 1998; Maeda et al., 2004) and the possibility of multi-drug resistance to kanamycin and ampicillin in *B. glumae* (Zhou-Qi et al., 2016).

Shifts in resistance and repeated disease outbreaks indicate that more research on bacterial mechanisms is needed to prevent lethal antibiotic effects. We previously reported that GluR, but not GluS, is involved in the control of cell division (Marunga et al., 2021). Therefore, in the present study, we investigated the effect of *gluS* and *gluR* null mutations on antibiotic sensitivity in *B. glumae* BGR1. Our findings provide new insight into antibiotic resistance mechanisms, and suggest a novel therapeutic approach for successful control of bacterial pathogens.

## Materials and Methods

### Bacterial Strains and Growth Conditions

Bacteria cells were grown at 37°C and 250 rpm in Luria Bertani (LB) medium containing 0.1% (w/v) tryptone, 0.5% (w/v) yeast extract, and 0.5% (w/v) sodium chloride, with 1.5% agar added when needed (Affymetrix, Cleveland, OH, United States) along with appropriate antibiotics. The bacterial strains and plasmids used in this study are listed in Supplementary Table S1. The following antibiotics were used: 100 μg/ml rifampicin, 10 μg/ml tetracycline, and 50 μg/ml kanamycin. The genetic information and gene IDs used in this study were obtained from the *B. glumae* BGR1 genome database (GenBank accession nos. CP001503–CP001508). We used previously developed TCS mutants and mutant complementation strains (Marunga et al., 2021).

### β-Lactam Susceptibility Test

A cell volume of 4 × 10^8^ cells/ml was transferred from overnight LB cultures into fresh LB broth and grown to the mid-log phase. An equal volume of cells was spread on LB agar supplemented with varying concentrations (25, 50, 75, 100, and 150 μg/ml) of β-lactam antibiotics (penicillin-G, ampicillin, and carbenicillin) according to the manufacturer’s instructions. Bacterial growth in antibiotic-free LB agar was quantified by direct counting using a colony-forming unit (CFU)-based method.

### Viability Assay

To supplement the susceptibility test, we performed a live/dead cell assay on bacterial cells exposed to 50 μg/ml of carbenicillin antibiotics for 0, 12, 24, and 36 h using the LIVE/DEAD BacLight Bacterial Viability Kit following the manufacturer’s instructions. The kit contains SYTO 9 fluorescent green nucleic acid stain and a red fluorescent nucleic acid stain [propidium iodide (PI); Invitrogen, Carlsbad, CA, United States]. Fluorescence images were captured using a confocal laser scanning microscope (SP8X; Leica, Wetzlar, Germany) at excitation/emission wavelengths of 483/490–540 and 535/890–680 nm for green and red fluorescence, respectively.

### β-Lactamase Activity Assay

β-lactamase activity was quantified through the hydrolysis of the chromogenic substrate nitrocefin (DAWIN_BIO_; Abcam, Cambridge, United Kingdom), as described previously (Li et al., 2016) with some modifications. Overnight LB cultures were subcultured in fresh LB medium for 36 h. After 12 h, the cells were harvested, washed, and dissolved in phosphate-buffered saline (PBS, pH 7.4), and then sonicated using the Vibra-Cell ultrasonic processor (Sonics & Materials Inc., Newtown, CT, United States). The cell lysate was incubated with 50 μg/ml of nitrocefin for 10 min at room temperature, and the OD_450_ was determined.

### Detection of Penicillin-Binding Proteins

Bacterial membranes harboring penicillin-binding proteins (PBPs) were prepared from LB – carbenicillin (50 μg/ml) cultures grown for 12 h. Uninhibited PBPs were labeled with a fluorescent penicillin (Bocillin FL; Invitrogen, Carlsbad, CA, United States) and quantified by measuring fluorescence intensity as described previously (Zhao et al., 1999). To observe PBP localization in the bacterial membrane, Bocillin FL-labeled cells were further subjected to a membrane dye, FM-6-64 (Invitrogen; Kocaoglu et al., 2012) and then observed using a confocal laser scanning microscope (SP8X; Leica) at excitation/emission wavelengths of 488/500–535 and 515/621–678 nm for Bocillin FL and FM-6-64, respectively.

### Quantitative Reverse-Transcription PCR

Total RNA was isolated from *B. glumae* strains using the RNeasy Mini Kit (Qiagen, Hilden, Germany) following the manufacturer’s instructions. cDNA was synthesized as described previously (Marunga et al., 2021) using Recombinant RNasin and M-MLV reverse transcriptase (Promega, Madison, WI, United States) according to the manufacturer’s instructions. The primer sets used in quantitative reverse-transcription PCR (qRT-PCR) are listed in Supplementary Table S2. Transcription levels were determined using SsoFast EvaGreen Supermix (Bio-Rad, Hercules, CA, United States) under the following thermal cycling conditions: 95°C for 30 s, followed by 30 cycles of 95°C for 5 s and 55°C for 5 s. All reactions were performed in triplicate and normalized to the 16S *rRNA* gene using CFX Manager software (Bio-Rad).

### Electrophoretic Mobility Shift Assay

For the electrophoretic mobility shift assay (EMSA), previously purified GluR-His (Marunga et al., 2021) was used. The promoter regions of the putative GluR target DNA were amplified using the primer sets listed in Supplementary Table S2. The resulting PCR products were labeled with biotin using LightShift Chemiluminescent Electrophoretic Mobility Shift Assay Kits (Pierce, Rockford, IL, United States), following the manufacturer’s instructions. As non-specific competitor DNA, a region 329 bp upstream of *katE1* was used, amplified using the primers KatE1-F/R (Supplementary Table S2). The purified GluR-His (1–2 μM) was incubated in binding buffer [10 mM Tris–HCl (pH 7.5), 100 mM NaCl, and 5% glycerol (v/v)] containing 2 nM of biotin-labeled DNA, as described previously (Kim et al., 2007). For the competition assay, unlabeled target DNA at 20-fold molar excess was added to each reaction mixture along with the labeled DNA. The reactions were separated using 4% (w/v) polyacrylamide gels and transferred to nitrocellulose membranes. The bands were detected using streptavidin/horseradish peroxidase-derived chemiluminescence kits (Pierce), following the manufacturer’s instructions, and images were visualized using ChemiDoc XRS+ and Image Lab software (Bio-Rad).

### Statistical Analyses

All experiments were conducted in triplicate with respective controls. One-way ANOVA followed by Tukey’s honestly significant difference (HSD) *post hoc* test were performed using SPSS software (ver. 25.0; IBM Corp., Armonk, NY, United States) to detect significant differences. Statistical significance was evaluated at the levels of *p* < 0.05, 0.005, and 0.0005. All figures were prepared using Adobe illustrator software (ver. 25.2; Adobe Inc., San Jose, CA, United States).

## Results

### Mutations in GluS-GluR TCS Were Associated With β-Lactam Antibiotic Resistance in *B. glumae*

**Figure 1:**
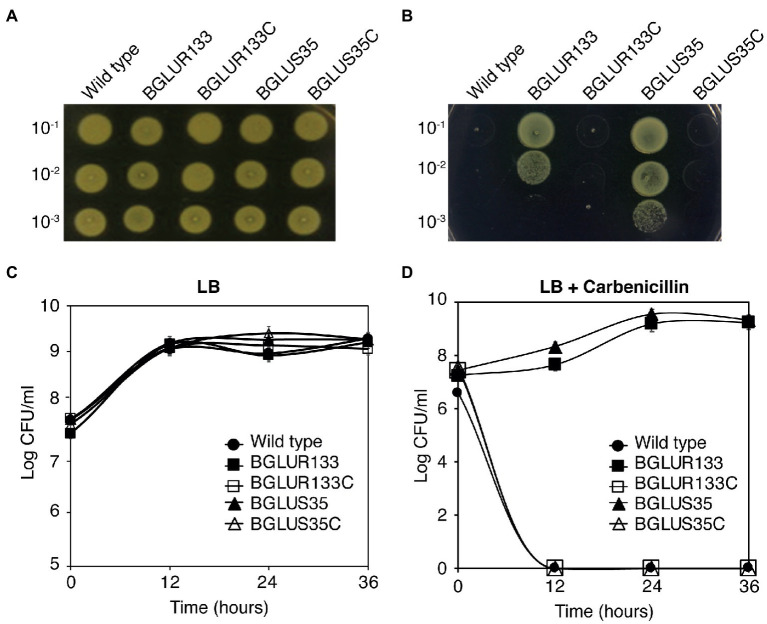
Mutations in the GluS-GluR two-component system (TCS) induce β-lactam antibiotic resistance in *Burkholderia glumae*. **(A,B)** Bacteria strains of the wild-type (WT), *gluS* mutant (BGLUS35), *gluR* mutant (BGLUR133), and complemented mutant strains (BGLUS35C and BGLUR133C) grown on plain Luria Bertani (LB; **A**) and LB medium with 50 μg/ml carbenicillin **(B)** were serially diluted and spotted on LB agar. The plates were incubated for 2 days at 37°C and then photographed. **(C,D)** Cell population density was quantified in the indicated bacteria strains in **(C)** LB and **(D)** LB containing carbenicillin by counting colony-forming units (CFUs). The results are expressed as logarithmic values of CFU/ml. Error bars denote SE of experiments performed in triplicate.

Since, we previously reported that GluR regulates the expression of cell division genes in *B. glumae*, we reasoned that this TCS might be involved in antibiotic sensitivity as well. To test this hypothesis, we examined the ability of the TCS null mutants to tolerate selected β-lactam antibiotics such as carbenicillin. Contrary to plain LB, the wild-type (WT) BGR1 experienced rapid cell death, whereas the *gluS* and *gluR* mutant strains (BGLUS35 and BGLUR133, respectively) showed sustained growth when spotted on LB supplemented with 50 μg/ml of carbenicillin (Figures 1A,B). We then assessed the minimum inhibitory carbenicillin concentrations of the TCS mutants using increasing carbenicillin concentrations. Both mutant strains tolerated a high concentration of carbenicillin (150 μg/ml; Supplementary Figure S1).

Furthermore, quantification of bacterial growth in liquid LB medium showed that both the mutant strains required approximately 12 h to adapt to the antibiotic environment, after which they grew and multiplied in a manner similar to those in the carbenicillin-free environment (Figures 1C,D). These results indicate that the BGLUR133 and BGLUS35 strains had acquired resistance against carbenicillin. Genetic complementation of *gluS* and *gluR* (BGLUS35C and BGLUR133C, respectively) restored β-lactam sensitivity to the strains (Figures 1A–D), confirming that the observed phenotypes were caused by mutations in the GluS-GluR TCS.

The BGLUR133 and BGLUS35 strains were also resistant to penicillin-G and ampicillin, whereas the WT BGR1 was not (Supplementary Figure S2).

### Cell Viability of *B. glumae* Strains in Response to β-Lactam Antibiotic Treatment

**Figure 2:**
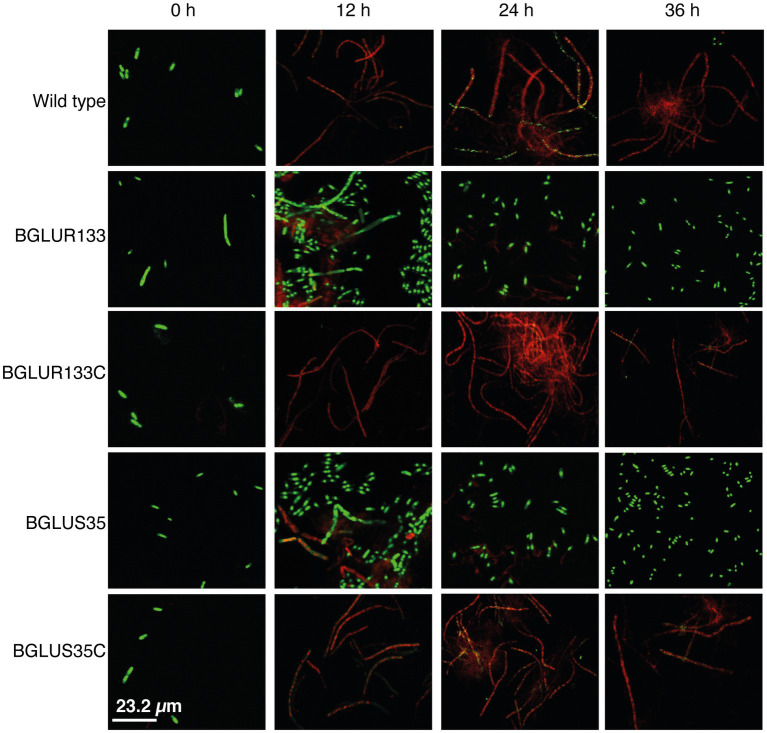
The *gluS* and *gluR* mutants maintained viability in a carbenicillin environment. Cell viability of the WT, TCS mutants (BGLUS35 and BGLUR133), and their respective mutant complemented strains (BGLUS35C and BGLUR133C) cultured in LB medium with 50 μg/ml carbenicillin was assessed by combination staining with propidium iodide (PI) and SYTO 9 green at the indicated times. Fluorescence images were obtained at excitation/emission wavelengths of 483/490–540 and 535/890–680 nm for green (live cells) and red (dead cells) fluorescence, respectively.

Since β-lactam antibiotics form osmotically fragile filamentous cells that are prone to cell lysis, we hypothesized that the *gluS* and *gluR* mutants would maintain normal cell division and form viable cells upon exposure to carbenicillin, despite the fact that *gluR*, but not *gluS*, formed filamentous cells in plain LB broth. To test this hypothesis, we assessed the morphology and viability of the WT and TCS mutants BGLUS35 and BGLUR133, alongside their respective complemented strains, BGLUS35C and BGLUR133C, using carbenicillin treatment. We performed a mixed stain assay, using green fluorescent SYTO 9 and red fluorescent PI nucleic acid to examine viable and dead cells, respectively, after 12 h of carbenicillin treatment. BGLUS35 and BGLUR133 formed heterogeneous cell cultures of both live (green) and dead (red) filamentous and normal rod-shaped cells, in contrast to the red fluorescent filamentous cells formed in the WT BGR1 (Figure 2). Interestingly, after 24 h of incubation, the mutant cultures were dominated by green fluorescent rod-shaped cells, whereas all filamentous cells appeared to have died (Figure 2). The phenotype susceptible to carbenicillin was restored by the complemented mutant strain (Figure 2), indicating that the altered GluS-GluR TCS triggered resistance to carbenicillin antibiotics in *B. glumae*.

### Increased β-Lactamase Activity in GluS-GluR TCS Mutants Led to Acquired Carbenicillin Resistance

**Figure 3:**
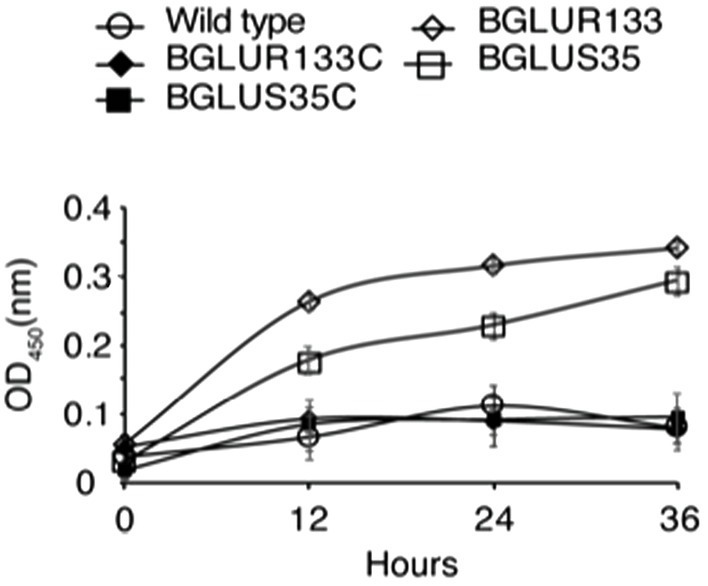
The role of GluS-GluR in the expression of β-lactamases. β-lactamase activity was measured in LB medium according to nitrocefin substrate hydrolysis, and relative amounts of β-lactamase produced by each strain quantified at OD_450_ at the indicated times. Data are mean ± SE of triplicate experiments. PBS, phosphate-buffered saline (pH 7.4).

To determine the cause of the observed resistance phenotype, we hypothesized that mutations in the GluS-GluR TCS might have triggered the production of β-lactam hydrolytic enzymes, i.e., β-lactamases. β-lactamase activity was assessed using nitrocefin, a β-lactamase substrate. We quantified the total β-lactamase produced by measuring the OD_450_ of the nitrocefin hydrolysates in LB medium. Higher OD_450_ values were recorded for the BGLUS35 and BGLUR133 strains (Figure 3) than for the WT, which decreased to near zero (Figure 3). The complemented BGLUS35C and BGLUR133C strains reinstated the inability to hydrolyze nitrocefin (Figure 3).

**Figure 4:**
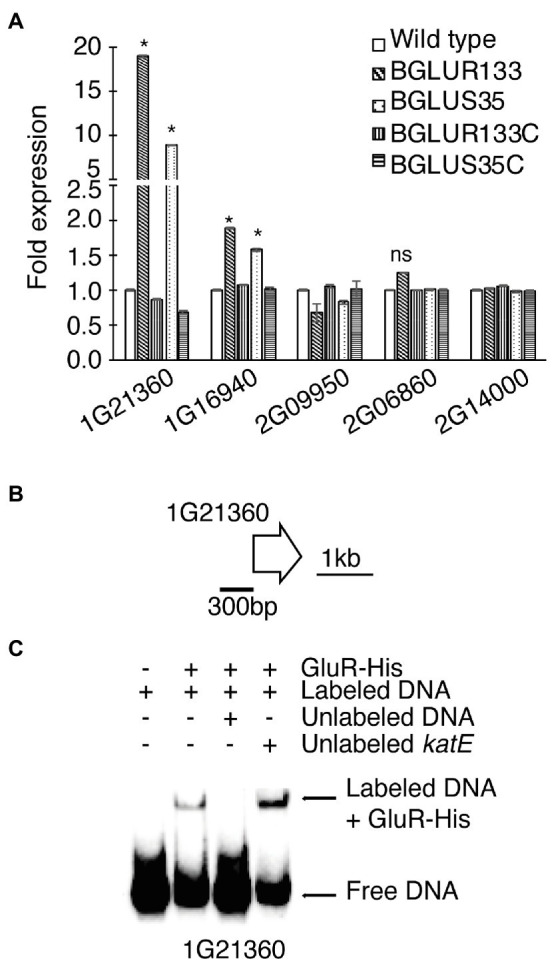
The β-lactamase expression levels in *B. glumae* BGR1 strains. **(A)** Quantitative reverse-transcription PCR (qRT-PCR) analysis of the indicated β-lactamase genes was conducted for the WT BGR1, *gluS* mutant (BGLUS35), *gluR* mutant (BGLUR133), and their respective complemented mutant strains (BGLUS35C and BGLUR133C). Data are mean ± SE of triplicate experiments. Statistical analysis was performed using one-way ANOVA followed by Tukey’s test for multiple comparisons (^*^p < 0.05; *F*_19,40_ = 3,924.655, *p* = 0.00). NS, not significant. **(B)** Gene map showing the putative promoter region of the metallo-β-lactamase (MBL) gene BGLU_1G21360 used in the electrophoretic mobility shift assay (EMSA). **(C)** EMSA results showing direct binding of GluR-His to the putative promoter region of BGLU_1G21360; 2 μM GluR-His, 2 nM labeled target DNA, 2 nM unlabeled *katE* non-competitor DNA, and 20 nM unlabeled target promoter DNA were used for EMSA.

According to genetic analyses, *B. glumae* BGR1 harbors four classes of β-lactamases, including β-lactamase class A protein (BGLU_2G14000), metallo-β-lactamase (MBLs) superfamily proteins (BGLU_2G09950, BGLU_1G16940, and BGLU_1G21360), class C protein (BGLU_2G06860), and class D protein (BGLU_2G15400). We attempted to identify the family involved in the increased β-lactamase activity. We performed qRT-PCR analysis and found significant increases in the expression of BGLU_1G21360 and BGLU_1G16940, encoding MBLs in BGLUS35 and BGLUR133, compared to the WT strain (Figure 4A). The complemented strains, BGLUS35C and BGLUR133C, were associated with reversion of MBL expression to levels comparable to those of the WT. The remaining β-lactamase classes were not significantly affected by GluS-GluR mutations (Figure 4A). We further evaluated whether GluR directly controls MBL gene expression using EMSAs with the putative promoter regions of BGLU_1G21360 and purified His-tagged GluR (GluR-His; Figure 4B). Direct binding of GluR-His to the putative promoter regions of BGLU_1G21360 showed that GluR directly regulates MBL expression (Figure 4C).

### BGLUS35 and BGLUR133 Showed Elevated PBP Gene Expression

**Figure 5:**
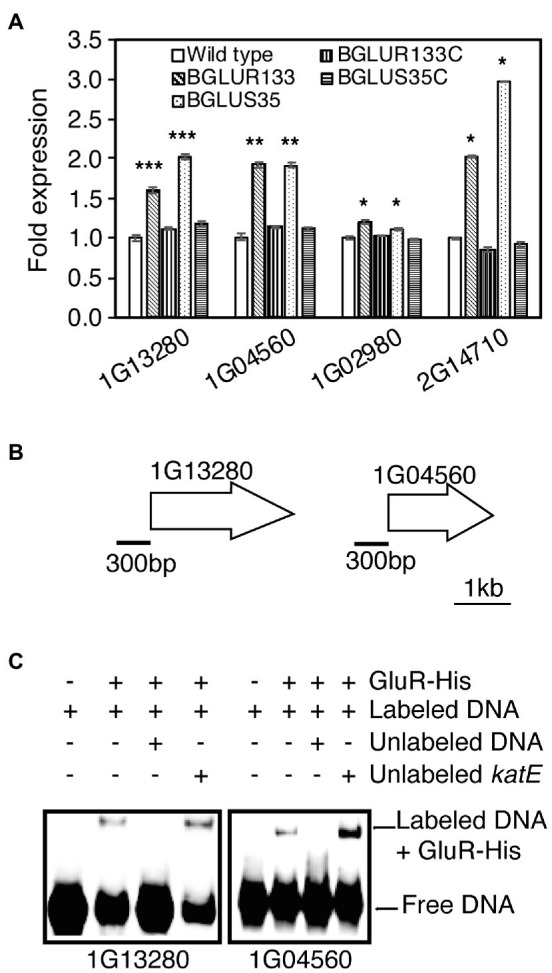
The role of GluS-GluR in the expression of penicillin-binding proteins (PBPs) in *B. glumae* strains. **(A)** qRT-PCR analysis of PBP gene expression in the indicated strains. Genes shown in the graph include BGLU_2G14710, BGLU_1G02980 (1A family), BGLU_1G04560 (2A family), and BGLU_1G13280 (membrane carboxypeptidase). Data are mean ± SE of triplicate experiments. Statistical analysis was performed using one-way ANOVA, followed by Tukey’s test for multiple comparisons (^***^p < 0.0005; ^**^p < 0.005; ^*^p < 0.0005; *F*_19,40_ = 1,550.875, *p* = 0.00). **(B)** Gene maps showing the putative promoter regions of the selected genes for EMSA. **(C)** EMSA results showing direct binding of GluR-His to the putative promoter regions of BGLU_1G13280 and BGLU_1G04560; 1 μM GluR-His, 2 nM labeled target DNA, 2 nM unlabeled *katE* non-competitor DNA, and 30 nM unlabeled target promoter DNA were used for EMSA.

Antibiotic-susceptible β-lactams form irreversible covalent bonds with PBPs, arresting the cell wall assembly process. Therefore, we reasoned that PBP gene expression might be elevated in the *gluS* and *gluR* mutants to produce more PBPs. To determine how GluS-GluR TCSs influence the expression of genes encoding PBPs, we quantified PBP gene expression in each strain using qRT-PCR analysis. The results showed that PBP expression levels were significantly higher in the TCS mutants than WT (Figure 5A). The complemented strains maintained low PBP levels comparable to those of the WT (Figure 5A). Next, we performed EMSAs to assess the ability of GluR-His to bind to the selected putative promoter regions of PBP genes, and found that GluR-His bound to the putative promoter regions of BGLU_1G13280 and BGLU_1G04560, indicating a repressive role of GluR for these genes (Figures 5B,C).

**Figure 6:**
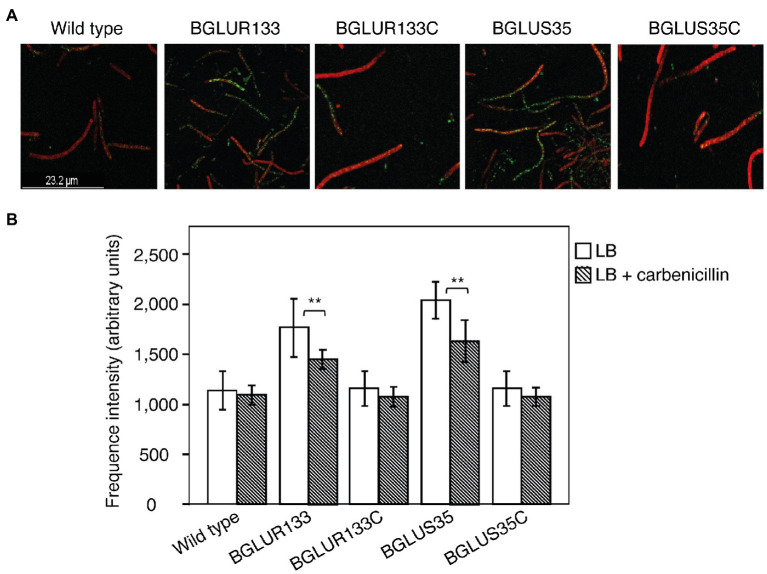
Characterization of PBPs in the indicated *B. glumae* strains. **(A)** Uninhibited PBPs cultured overnight in LB containing 50 μg/ml carbenicillin were stained with Bocillin FL and then subjected to a cell membrane dye (FM-4-64). Green fluorescence spots indicate uninhibited PBPs. **(B)** Quantification of PBP fluorescence intensity using Bocillin FL. Fluorescence images and intensity values were obtained at excitation/emission wavelengths of 488/500–535 and 515/621–678 nm for Bocillin FL and FM-6-64, respectively. Data are mean ± SE of triplicate experiments. Statistical analysis was performed using one-way ANOVA, followed by Tukey’s test for multiple comparisons (^**^p < 0.05; *F*_4,20_ = 31.455, 31.506, *p* = 0.00 for LB and LB with carbenicillin, respectively).

Then, we applied Bocillin FL to visualize and quantify uninhibited PBPs after exposure to carbenicillin antibiotics. The BGLUS35 and BGLUR133 strains contained significant numbers of uninhibited PBPs, as indicated by higher green fluorescence intensity compared to the attenuated levels observed in the WT strain (Figures 6A,B). The complemented strains, BGLUS35C and BGLUR133C, responded in a similar manner to the WT (Figures 6A,B).

## Discussion

β-lactams affect bacterial cell wall assembly by covalently modifying PBP active sites (Park and Strominger, 1957). This alteration inhibits enzyme activity, damages the integrity of the cell wall, and leads to cell lysis (Park and Strominger, 1957; Cho et al., 2014). Although β-lactams are among the most effective antibiotics, they are often challenged by antibiotic resistance. Among the various mechanisms by which bacteria acquire antibiotic resistance, we identified unexpected roles of TCSs in β-lactam antibiotic resistance in *B. glumae*, whereas the TCSs of other bacteria have been reported to play positive roles in acquiring antibiotic resistance (Arthur et al., 1992; McPhee et al., 2006; Hong et al., 2008; Gooderham and Hancock, 2009; Zamorano et al., 2014; Li et al., 2016). In contrast, the presence of GluS-GluR TCSs conferred sensitivity to β-lactam antibiotics in *B. glumae*. Since mutations in the *Staphylococcus aureus* WalK-WalR TCS have been shown to induce resistance to multiple drugs, including vancomycin and daptomycin (Howden et al., 2011), it would be unsurprising for similar roles to be discovered in the TCSs of other bacteria.

The production of osmotically fragile filamentous cells prone to lysis, and ultimately cell death, is a consistently observed feature of the β-lactam antibiotic effect (Kong et al., 2010). Analysis of the WT BGR1 at the onset of carbenicillin treatment revealed similar phenotypes. Although the WT was entirely inhibited, only filamentous cells in the mutant strains were susceptible to antibiotics, leaving behind a homogeneous culture of normal, viable rod-shaped cells in *gluS* and *gluR* mutant cultures. Considering that the *gluS* mutant forms normal cells, unlike the filamentous cells of the *gluR* mutant, in LB without carbenicillin treatment (Marunga et al., 2021), we conclude that the filamentous cell formation observed in both the *gluS* and *gluR* mutants was caused by carbenicillin treatment, as observed in WT cells. Therefore, we hypothesized that mutations in GluS-GluR switched on the resistance mechanism toward β-lactams. The short-lived filamentous cells may have resulted from the adaptation period, as the strains were “rewired” for an appropriate response.

The results of this study raise the question of how mutations in the GluS-GluR TCS cluster impact the function of the regulator leading to β-lactam resistance. β-lactam resistance is associated with the production or induction of β-lactamases, target site reinforcement in PBPs, and transport channel modification (Wilke et al., 2005). This study discovered that mutations in the GluS-GluR cluster induced nascent turnover of β-lactamase that significantly improved hydrolytic activity in *B. glumae*. Increased β-lactamase production in the TCS mutants suggests increased resistance to the antibiotics. β-lactamases are grouped into four distinct classes (A–D) based on their DNA sequences and catalytic requirements (Poole, 2004; King et al., 2017). Classes A, C, and D comprise metal-independent enzymes that utilize an active site serine to achieve hydrolytic activity (King et al., 2017). Class B enzymes, also known as MBLs, require zinc ions to facilitate opening of the β-lactam ring (King et al., 2017). The secretion and zinc ion dependency of β-lactamase are important for β-lactam resistance in bacteria (King et al., 2017), and extracellular zinc ion-dependent MBLs degrade all groups of β-lactam antibiotics except monobactams (Walsh et al., 2005; Sacha et al., 2008). Among the annotated MBLs in *B. glumae*, an MBL encoded by BGLU_1G21360 was found to be responsible for improved hydrolytic activity in the *gluS* and *gluR* mutants. However, whether this MBL is secreted and dependent upon zinc ions remains unknown.

β-lactamase induction was previously considered an indirect response to β-lactam-mediated cell wall damage (Hofer, 2016). However, bacteria have evolved over time and now adopt less costly defense mechanisms, such as exploiting TCSs that sense antibiotics and direct an appropriate response without damaging cell wall integrity. A good example is the enteric pathogen *Vibro parahaemolyticus*, in which a conserved VbrK-VbrR TCS was reported to regulate the expression of β-lactamases in response to β-lactam exposure; mutations in this TCS yielded β-lactam-susceptible strains (Li et al., 2016). Despite our contrasting findings, we suggest that more β-lactamases were produced in the *gluR* and *gluS* mutants than in the WT BGR1, without damaging cell wall integrity. This mechanism would explain how mutations in the GluS-GluR TCS confer resistance against β-lactam antibiotics without damaging the cell wall.

The inhibition of PBPs, which disrupts the equilibrium between peptidoglycan (PG) synthases and the cell matrix-expanding action of PG hydrolases, is widely accepted as the mode of action of β-lactams (Tomasz, 1979). The expression of genes encoding PBPs was significantly higher in β-lactam-resistant BGLUS35 and BGLUR133 than in the WT, which was not unexpected. The presence of large numbers of uninhibited PBPs after carbenicillin treatment further confirmed the β-lactam resistance phenotypes of the TCS mutants. This finding suggests that PBP overexpression allowed the mutants to overcome carbenicillin treatment and guaranteed continued cell division in the antibiotic environment. A similar mechanism was proposed for *S. aureus* to explain how bacteria gain antibiotic resistance (Wu et al., 2001; Sauvage et al., 2002; Wilke et al., 2005). This bacterium exerted resistance against β-lactam antibiotics through the generation of β-lactam-insensitive PBPs (Wu et al., 2001) or increasing PBP expression levels (Sauvage et al., 2002; Wilke et al., 2005). Our findings are similar to the latter case; it would not be surprising to discover additional similar cases in other bacteria. To support the notion that GluR is directly involved in the expression of genes for β-lactam antibiotic resistance, we identified a conserved inverted repeat sequence in the upstream sequences of MBL (BGLU_1G21360) and PBPs (BGLU_1G13280 and BGLU_1G0456). These sequences were comparable to those previously proposed as possible GluR binding sites (Marunga et al., 2021; Supplementary Figure S3); however, further research is needed to confirm this. Furthermore, techniques such as RNA-seq analysis would be recommended to comprehensively investigate other putative GluR and GluS targets in β-lactam antibiotic resistance. While this study focused on the response of GluS and GluR in β-lactam antibiotic resistance, it would also be interesting to investigate the response of this TCS cluster in other antibiotics families such as quinolones.

Although multiple cellular targets have previously been proposed as ideal candidates for minimizing the incidence of mutational resistance (Silver, 2011), our findings demonstrate that bacteria can devise various resistance mechanisms to counteract drug effects. Therefore, our study calls into question the benefits of multicellular targeted therapies that rely on the assumption that bacteria cannot create mutations that trigger multiple resistance mechanisms. Thus, the use of a central system as a therapeutic target, such as TCS, may lead to stronger resistance in bacteria, by allowing them to hide behind therapeutic targets and keep potential drug resistance mechanisms in reserve. Although GluS-GluR mutations improve the fitness of *B. glumae* in a β-lactam environment, they also jeopardize bacterial cell division (Marunga et al., 2021). It is important to discover the evolutionary processes of this bacterium following exposure to various environments, since GluS-GluR TCS mutations cause various phenotypic changes that affect fitness in nature.

## Data Availability Statement

The original contributions presented in the study are included in the article/Supplementary Material; further inquiries can be directed to the corresponding author.

## Author Contributions

JM and IH designed the experiments, contributed reagents, materials, and analysis tools, and wrote the paper. JM and EG performed the experiments. JM, EG, YK, and IH analyzed the data. All authors contributed to the article and approved the submitted version.

## Conflict of Interest

The authors declare that the research was conducted in the absence of any commercial or financial relationships that could be construed as a potential conflict of interest.

## Publisher’s Note

All claims expressed in this article are solely those of the authors and do not necessarily represent those of their affiliated organizations, or those of the publisher, the editors and the reviewers. Any product that may be evaluated in this article, or claim that may be made by its manufacturer, is not guaranteed or endorsed by the publisher.
