# Supplementary material for: Mutations in the Two-Component GluS-GluR Regulatory System Confer Resistance to β-Lactam Antibiotics in Burkholderia glumae
Source: Front Microbiol. 2021 Jul 26;12:721444. doi: 10.3389/fmicb.2021.721444 (PMC8350040; doi:10.3389/fmicb.2021.721444)
Supplement: Supplementary file 1 [file Data_Sheet_2.pdf]

Supplementary Material

Figure S1. β-lactam tolerance in *Burkholderia glumae* strains induced by carbenicillin antibiotics. GluS-GluR mutant strains tolerated up to 150 µg/m1 of carbenicillin.

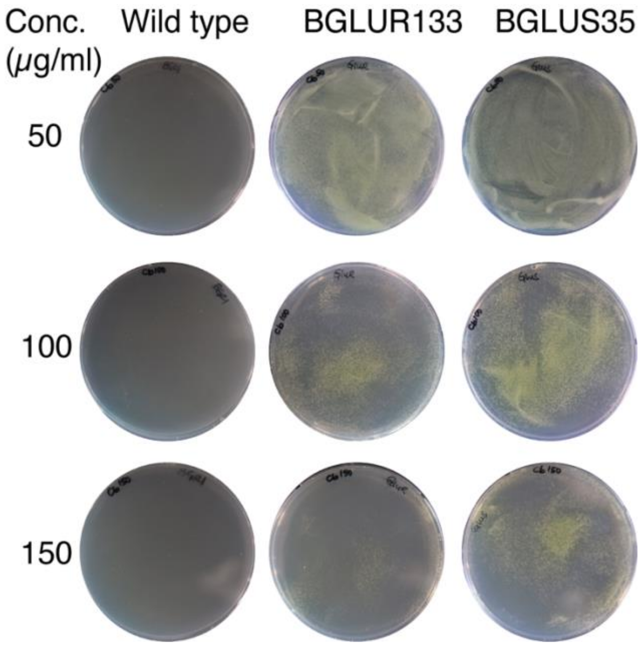

Figure S2. Responses of the *B. glumae* strains to ampicillin and penicillin-G β-lactam antibiotics.

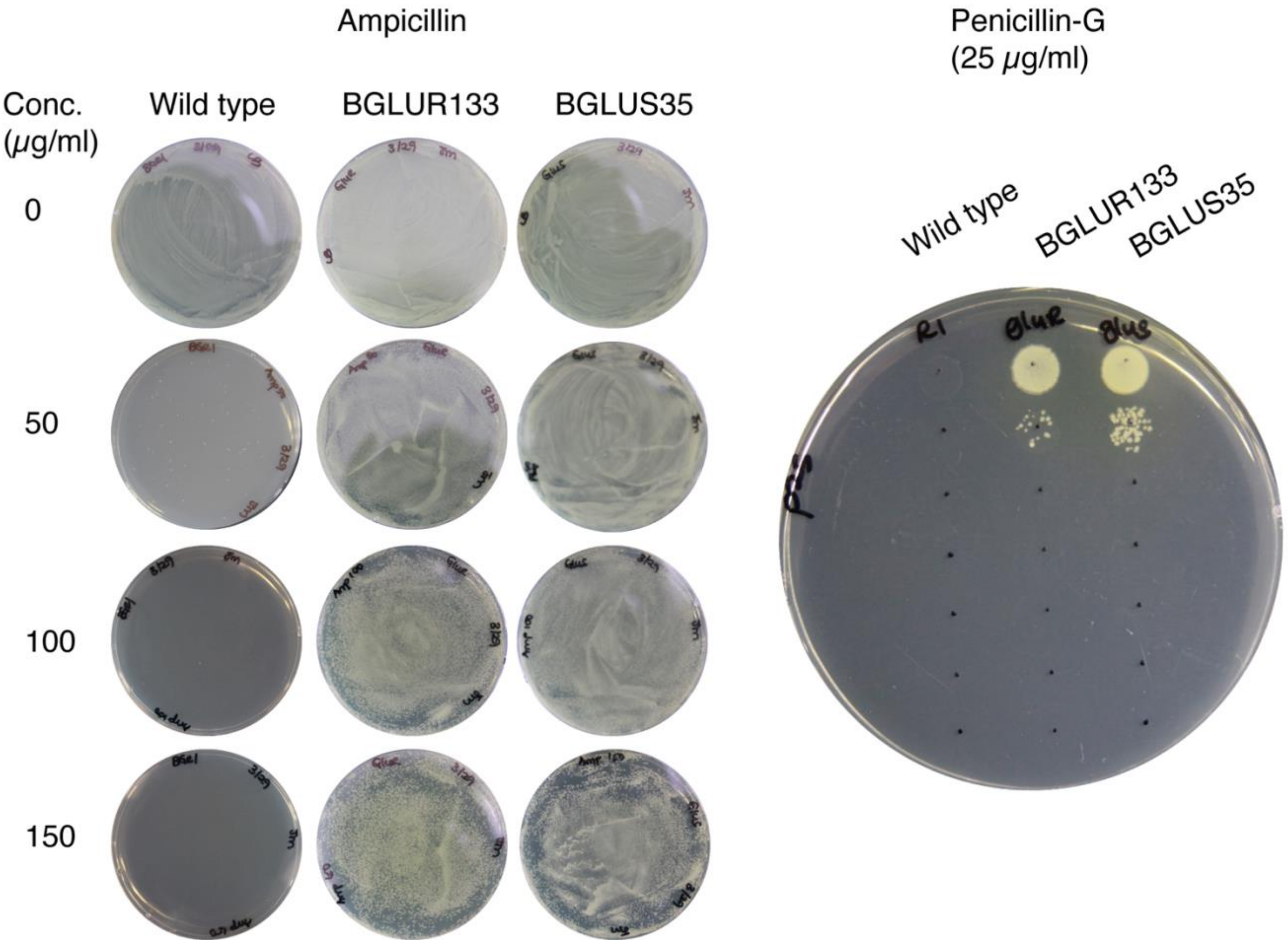

Figure S3. Upstream sequences of metallo-β-lactamases (MBLs) (BGLU_1G21360), PBP1 (BGLU_1G13280), and PBP2A (BGLU_1G04560), which were used in the electrophoretic mobility shift assay (EMSA). Red underlining indicates conserved inverted repeat sequences; black underlining indicates potential ribosome binding sites within the sequences. Inverted repeat sequences are indicated by arrows; conserved regions within the three genes are highlighted.

MBL (BGLU_1G21360)

GCTGCCGGTCGCGCTGCGTGCCTTCGTGGCGCAGGATTTCTCGATGTGGGAGGCGATCCT
GCGCCAGCTCGGCGTGCCGAACGGCGCGCAGATCGTGCGCGAGTGCCGCCAGCCCGGC
AGCGGCAACACGCTGACCCGCGACTACAGCGGGCTGCACCGGCTCGCCGACGAGCTCG
GCATCCTCGGGCAACTCGATTTCGAGTTCGCCGGCACCACGCGGATCGCCCATTGAGCG
CCGCCCGCCAGCCCCCGAGGTCCATCATGCAACCAGCTTCAGGGATTTCTCAG**ATG**

PBP1 (BGLU_1G13280)

CCTTCTCGCTTCCGATGATAGCGGGCGTGGGTGACGGTGGCGCGTCGGCGTGGCGGTGG
CGGGCGCGTTGCGCGGCGCGCGTGCCCCGCGATGGGGCAGGCAGCGGGAATTTTTACAA
AAAATCGCGTGGGGTGCCGGACGATTTCATTAAAATGCGCTGTCGCGTCGCGGGAATGG
ATCGCCGCGTGCCGTGATCCGGTGATGTGCTACGCTCCTGCGCATCGCCTCAAGCCCCAA
CACCTAAGATCCACACGCAC**ATG**

PBP2A (BGLU_1G04560)

ATCAGCAGCGGCAGATCTTCTTCCAGTTGCAGCGTGCCCAGTCGCAGGAGCATCAGCTC
CAGCAGGACTACGCGCAGCTGCAGTATCAACAGAGCGCGTTGTCGAAGACCTCGCGCAT
CGAGCAGTTGGCCAACGATTCGCTGAAGATGCAGCCGATCGCCACCGGCCGCACCCAAT
ACCTGGTGCTGGCGCCGGGCGCCGCCAAGGCCGTCGATGCGCCGCTGCCCACCTCGGCC
GCGTCGGGCTCGAAGGGGAGCGCGCG**ATG**

B. glumae BGR1 MBL: CGCGCAGATCGTGCGCG
B. glumae BGR1 PBP1: GGCGCGTTGCGCGG
B. glumae BGR1 PBP1: TGCGCTGTCGCGT
B. glumae BGR1 PBP2A: AGCGCGTTGTCGAAGACCTCGCGCA

Table S1. Bacteria strains used in this study

Burkholderia glumae	Characteristics	Source
BGR1	Wild type, Rif^R	Kim et al., 2004
BGLUR133	BGR1 *gluR*::Tn*3-gusA133*, Km^R	Marunga et al., 2021
BGLUS35	BGR1 *gluS*::Tn*3-gusA35*, Km^R	Marunga et al., 2021
BGLUR133C	BGR1, *gluR*::Tn*3-gusA133* containing pBGH13, Km^R, Tet^R	Marunga et al., 2021
BGLUS35C	BGR1, *gluS*::Tn*3-gus35* containing pBGH13, Km^R, Tet^R	Marunga et al., 2021
pGluR-His	*gluR* in pET21b, Amp^R	Marunga et al., 2021

Rif^R, rifampicin resistance; Tet^R, tetracycline resistance; Km^R, Kanamycin resistance; Amp^R; ampicillin resistance

Table S2. List of oligonucleotides used in this study (Purchased from Macrogen, Korea).

Primers	Sequence (5’ to 3’)
16S RNA-F	AGCCGCGGTAATACGTAGG
16S RNA-R	ACTCTAGCCTGCCAGTCACC
PBP1A-F	CTCGTAGTCCATCAGGCCG
PBP1A-R	CGCGCAATACAAGGACGAAA
PBP1-F	CGAGGCTATCGACGACGC
PBP1-R	CCACGAACTGCACCTCCAC
PBP2-F	ATCAGGAATACGGCATCGGC
PBP2-R	GCGACATCGACAGCCCATAG
PBP2A-F	ACATCACCTACGCCAACCRG
PBP2A-R	CGGGTAATTGACGAGCGAGA
bmlA-F	TTGTCGTATCCGGCGTCCT
bmlA-R	ATGCTCGGCAATACCACCG
MBL1 -F	GAGGACAGCCGGCCATTCT
MBL1-R	CTGATCCTGATCGAGCCCGTG
MBL2-F	GTGGTGCCGTCGAGTGTG
MBL2-R	TGGATCAACGACGAACCCAG
AmpC-F	GCCAGACATTTCGGCTCTTTC
AmpC-R	GTTCGAATCGGCACCTTTCC
1g21360p-F	GCGGCGTCGGGGCAGGCATC
1g21360p-R	GCTGCCGGTCGCGCTGCGTG
1g13280p-F	CCTTCTCGCTTCCGATGATA
1g13280p-R	TGGCGGGCGCTGCGGCGACA
1g04560-F	ATCAGCAGCGGCAGATCTTC
1g04560-R	CTCGACCCGAACTTGACGTT
KatE1-F	ACTCGCGCCGCTCGTCGAA
KatE1-R	CATCGGCATCCTGGGTCGC

F- forward primer; R – reverse primer

SUPPLEMANTAL REFERENCES

Kim, J., Kim, J.G., Kang, Y., Jang, J.Y., Jog, G.J., Lim, J.Y., et. al. (2004). Quorum sensing and the LysR-type transcriptional activator ToxR regulate toxoflavin biosynthesis and transport in *Burkholderia glumae*. *Mol. Microbiol.* 54**,** 921-934. doi: 10.1111/j.1365-2958.2004.04338.x

Marunga, J., Goo, E., Kang, Y., and Hwang, I. (2021). Identification of a Genetically Linked but Functionally Independent Two-Component System Important for Cell Division of the Rice Pathogen *Burkholderia glumae*. *Front. Microbiol.* In press. doi: 10.3389/fmicb.2021.700333
